# Computational analysis, alignment and extension of analogue series from medicinal chemistry

**DOI:** 10.2144/fsoa-2022-0033

**Published:** 2022-06-28

**Authors:** Atsushi Yoshimori, Jürgen Bajorath

**Affiliations:** 1 Institute for Theoretical Medicine, Inc., 26-1 Muraoka-Higashi 2-chome, Fujisawa, Kanagawa, 2510012, Japan; 2 Department of Life Science Informatics & Data Science, B-IT, LIMES Program Unit Chemical Biology & Medicinal Chemistry, Rheinische Friedrich-Wilhelms-Universität, Friedrich-Hirzebruch-Allee 5/6, Bonn, D 53115, Germany

**Keywords:** analogue design, analogue series, compound optimization, deep learning, SAR transfer, structure–activity relationships (SARs)

In medicinal chemistry, the generation and optimization of active compounds plays a central role [1,2]. Hit-to-lead and lead optimization (LO) efforts produce series of structural analogues used to explore structure–activity relationships (SARs), identify molecular regions most important for specific ligand-target interactions, and increase compound potency. During optimization, SAR progression is monitored. During later LO stages, potency and other optimization-relevant properties (such as solubility, metabolic stability or toxicity) must be improved in concert and balanced until a suitable candidate compound is obtained.

## Analogue series

An analogue series (AS) consists of compounds that share a conserved core structure and are distinguished by different substituents (R-groups) introduced at one or more sites. Typically, one AS is investigated at a time. For high-profile projects, different late-stage series might be developed in parallel to prioritize primary and back-up candidates for further preclinical and clinical assessment. Successful ASs proceeding from initial hit-to-lead efforts through the entire LO pipeline might ultimately contain hundreds of compounds. However, progress in compound optimization is far from being certain. Work on given ASs must often be discontinued because unsurmountable roadblocks are encountered such the presence of ‘flat’ SARs that are resistant to further optimization or compound toxicity that cannot be mitigated. In such cases, alternative active compounds (hits) are selected as starting points for optimization and new ASs are generated.

## Analogue design

In the practice of medicinal chemistry, the key question during any hit-to-lead or LO campaign is which analogue(s) to generate next. The decision process is strongly influenced by the knowledge and experience of medicinal chemists. Conventional R-group tables continue to represent the primary data structure for documenting and monitoring evolving ASs. Although LO is typically driven by knowledge and consideration of synthetic criteria, analogue design is also supported by computational approaches. For many years, quantitative SAR (QSAR) methods for predicting the potency of new analogues using linear or non-linear models have been among the most widely used computational methods in medicinal chemistry [3,4]. QSAR analysis covers a wide spectrum of approaches, ranging from simple manually generated decision trees [5] to topical machine-learning approaches [6], and is often used in combination with other computational methods for hit identification [6]. In addition, LO is supported through experimental determination of structures of analogue-target complexes and structure-based drug design [7,8].

Going beyond QSAR analysis, surprisingly little has been done so far to computationally investigate ASs from other viewpoints or design analogues in different ways. In the following, currently available approaches and new developments are discussed.

## Assessing progress in lead optimization

In addition to deciding which analogues to generate next, evaluating LO progress is another central task in practical medicinal chemistry, which is also strongly influenced by subjective assessment and expectations. Notably, recognizing the lack of sufficient progress during LO is as important as reaching milestones. However, judging when it might be time to finally discontinue work on a given AS is a difficult task and terminating a project a hard decision to take, especially when much work has already been invested.

From a methodological point of view, monitoring progress during LO in an objective manner is far from being trivial, given the diversity of projects and the typical focus on individual ASs. However, a few computational approaches have been introduced to evaluate SAR progression for evolving ASs [9–12]. For the most part, the underlying concepts are statistical in nature aiming, for example, to identify compounds during LO that are decisive for SAR progression and most informative [9]. Furthermore, chemical saturation and SAR progression analysis has been combined to estimate how likely it might be to further advance evolving ASs by generating additional analogues [12]. Such diagnostic computational approaches are capable of providing decision support during LO beyond subjective assessment from a more global perspective, which principally distinguishes them from activity prediction methods.

## Identification of analogue series

The predominant focus on individual ASs during LO is hardly motivating systematic analysis and comparison of ASs for given targets or across different targets. Such efforts go beyond single projects and require additional resources. However, the large number of compounds from medicinal chemistry that are becoming publicly available provides an important resource and knowledgebase for SAR analysis and compound optimization that should be taken into consideration. In the pharmaceutical industry, it is increasingly being recognized that complementing internal projects with external data and knowledge provides opportunities that should not be missed. The methodological framework for the systematic extraction of ASs from compound collections is available. For a given core structure, all analogues containing this core can be easily identified in databases via simple substructure searching. Furthermore, for a given compound, analogues can be obtained by searching for matched molecular pairs (MMPs) [13], which are defined as pairs of compounds that are only distinguished by a chemical modification of a single site. The MMP search is facilitated by fragmentation of exocyclic single bonds in compounds and sampling of resulting core structures and substituents [13]. MMP fragmentation also provides the basis for the systematic identification of ASs with single substitution sites and their structural organization in matrices reminiscent of R-group tables using the SAR matrix approach [14]. Furthermore, ASs with single or multiple substitution sites can also be systematically extracted from compound databases based on decomposition of compounds according to retrosynthetic rules and identification of common cores with varying substitution sites [15].

Hence, applying such algorithms, large numbers of ASs with activity against different targets can be obtained, providing a valuable resource for SAR exploration and compound optimization efforts. As further discussed below, systematic AS identification also enables other applications.

## Extension of analogue series

With the advent of deep machine learning and other artificial intelligence approaches in medicinal chemistry [16], new opportunities for compound design arise, especially through generative modeling [17]. Among deep learning architectures used for generative modeling are chemical language models that were adopted from the field of natural language processing [17,18]. For example, we generated such models to further expand the analogue design capacity of the SAR matrix approach [14]. Another chemical language model was specifically constructed for the iterative extension of ASs [19]. Therefore, more than 100,000 ASs with single substitution sites and activity against more than 2000 different targets were algorithmically extracted from public medicinal chemistry compounds. Analogues forming each AS were ordered according to increasing potency and the chemical language model was trained on R-group sequences of most of the potency-ordered ASs (excluding test sets) to predict R-groups of new analogues for series extension. Following principles from natural language processing, the chemical language model prioritized new R-groups based upon conditional probabilities derived from R-group sequence information. Because all sequences used to train the model followed ascending potency gradients, AS extension was implicitly directed toward R-groups likely to yield analogues with further increased potency. Hence, this approach to AS extension was devised as a conceptually novel alternative to QSAR predictions. In test calculations, the AS-based chemical language model reproduced potent analogues for many different series with high frequency, indicating significant potential for prospective applications [19].

## SAR transfer

**Figure 1.**
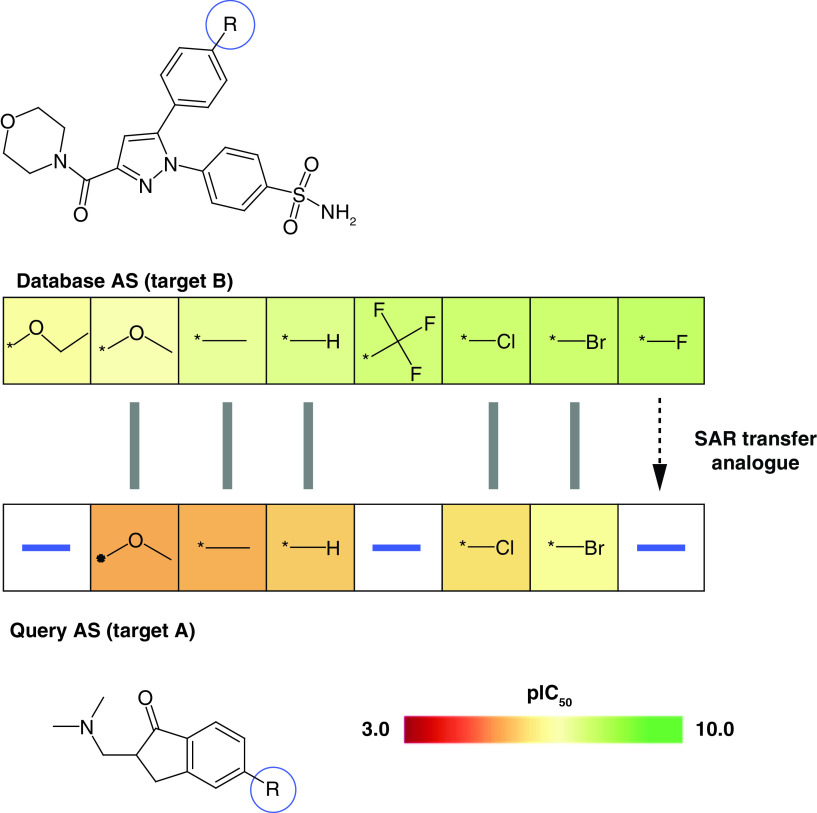
Structure–activity relationship transfer event. Shown is an exemplary alignment of a query AS with activity against target A (bottom) and a database AS active against another target B (top). Compounds in both ASs are arranged in the order of increasing potency. Hence, the alignment represents an SAR transfer event. For each AS, the core structure is shown and the substitution site (-R) is encircled in blue. Aligned analogues are indicated by grey bars. Cells containing R-groups of analogues are color-coded by compound potency (negative logarithmic IC_50_ values) according to the continuous color spectrum. The database AS contains a potent fluoro analogue (right) that is absent in the query AS. Accordingly, the fluoro derivative is suggested as an SAR transfer analogue for the query AS. AS: Analogue series; SAR: Structure–activity relationship.

If work on an AS needs to be discontinued during LO despite promising SAR progression (e.g., because of emerging toxicity) one would like to consider alternative core structures and corresponding analogues that might yield similar SAR trends. In other words, one would like to transfer an SAR from one series to another. This can be attempted computationally by searching for ASs with corresponding analogues having similar potency progression. For given targets, such SAR transfer events have been identified previously [20]. While target-based SAR transfer can be expected, in particular, for ASs with closely related core structures, an open question has been whether SAR transfer might also occur across different targets. To address this question, we have recently developed a methodology to systematically search for and align ASs with SAR transfer potential [21]. The approach follows principles of biological sequence alignment using dynamic programming. ASs are aligned based upon a chemical similarity matrix specifically generated for substituents. Potency-based ordering of ASs, as described above, ensures that meaningful alignments reveal ASs with corresponding analogues and increasing potency, hence meeting SAR transfer criteria. The methodology was applied to search a sample of potency-ordered test ASs against the remainder of the large pool of potency-ordered ASs [21]. Suitable alignments of ASs with activity against different targets were detected with high frequency, thus providing proof-of-principle for SAR transfer across different targets. ASs involved in SAR transfer often contained distinct core structures. Figure 1 shows a representative example. In addition to revealing SAR transfer, the alignments also provide suggestions for analogue design. If aligned database ASs contain highly potent analogues with substituents that are not present in the query AS, ‘SAR transfer analogues’ can be predicted as new candidates for a query AS, as illustrated in Figure 1. Such predictions are readily comprehensible from a medicinal chemistry perspective. In collaborative applications of SAR transfer analysis across different targets, potent SAR transfer analogues have been identified.

## Conclusion

In medicinal chemistry, compound optimization and AS generation play a central role. Although very large numbers of ASs can currently be extracted from public domain compounds, systematic analyses of ASs including the exploration of SAR transfer events have thus far been rare. This might at least in part be attributed to the prevalent single-series focus in the practice of medicinal chemistry. Only few studies have investigated ASs from a more global point of view. To these ends, algorithms for the systematic extraction of ASs from compound collections are essential. Although computationally identified ASs are detracted from a specific project context (for example, they contain no information about temporal analogue succession), these series provide a wealth of SAR information and an invaluable resource for LO projects. To complement LO efforts, computational predictions have long concentrated on standard QSAR approaches. However, as discussed herein, there are more opportunities to computationally support LO. These include diagnostic approaches for the assessment of SAR progression or chemical saturation of ASs and emerging deep learning schemes, which enable novel applications for AS extension, as exemplified by chemical language models. Hence, computational exploration of ASs in combination with predictive modeling will provide many exciting opportunities for future research. Hopefully, recent developments will entice more investigators to study ASs using different computational approaches and bridge between theoretical analysis, predictions and practical applications in medicinal chemistry.
